# Genome-wide association analysis identifies three new risk loci for gout arthritis in Han Chinese

**DOI:** 10.1038/ncomms8041

**Published:** 2015-05-13

**Authors:** Changgui Li, Zhiqiang Li, Shiguo Liu, Can Wang, Lin Han, Lingling Cui, Jingguo Zhou, Hejian Zou, Zhen Liu, Jianhua Chen, Xiaoyu Cheng, Zhaowei Zhou, Chengcheng Ding, Meng Wang, Tong Chen, Ying Cui, Hongmei He, Keke Zhang, Congcong Yin, Yunlong Wang, Shichao Xing, Baojie Li, Jue Ji, Zhaotong Jia, Lidan Ma, Jiapeng Niu, Ying Xin, Tian Liu, Nan Chu, Qing Yu, Wei Ren, Xuefeng Wang, Aiqing Zhang, Yuping Sun, Haili Wang, Jie Lu, Yuanyuan Li, Yufeng Qing, Gang Chen, Yangang Wang, Li Zhou, Haitao Niu, Jun Liang, Qian Dong, Xinde Li, Qing-Sheng Mi, Yongyong Shi

**Affiliations:** 1 Shandong Gout Clinical Medical Center, Qingdao 266003, China; 2 Shandong Provincial Key Laboratory of Metabolic Disease, The Affiliated Hospital of Qingdao University, Qingdao 266003, China; 3 Bio-X Institutes, Key Laboratory for the Genetics of Developmental and Neuropsychiatric Disorders (Ministry of Education), Shanghai Jiao Tong University, Shanghai 200030, China; 4 Institute of Social Cognitive and Behavioral Sciences, Shanghai Jiao Tong University, Shanghai 200240, China; 5 Department of Rheumatology and Immunology of the Affiliated Hospital, North Sichuan Medical College, Nanchong 737000, China; 6 The Affiliated Huashan Hospital of Fudan University, Shanghai 20040, China; 7 Henry Ford Immunology Program, Henry Ford Health System, 1 Ford Place, Detroit, Minnesota 48202, USA; 8 Department of Endocrinology, Fujian Provincial Hospital, Fujian Medical University, Fuzhou 350001, P.R. China; 9 Department of Dermatology, Henry Ford Health System, 1 Ford Place, Detroit, Michigan 48202, USA; 10 Department of Internal Medicine, Henry Ford Health System, 1 Ford Place, Detroit, Michigan 48202, USA; 11 Department of Urology, The Affiliated Hospital of Medical College Qingdao University, Qingdao 266003, China; 12 Department of Oncology, The Affiliated Hospital of Qingdao University Medical College, Qingdao 266003, China; 13 Department of Pediatric Surgery, The Affiliated Hospital of Qingdao University Medical College, Qingdao 266003, P.R. China

## Abstract

Gout is one of the most common types of inflammatory arthritis, caused by the deposition of monosodium urate crystals in and around the joints. Previous genome-wide association studies (GWASs) have identified many genetic loci associated with raised serum urate concentrations. However, hyperuricemia alone is not sufficient for the development of gout arthritis. Here we conduct a multistage GWAS in Han Chinese using 4,275 male gout patients and 6,272 normal male controls (1,255 cases and 1,848 controls were genome-wide genotyped), with an additional 1,644 hyperuricemic controls. We discover three new risk loci, 17q23.2 (rs11653176, *P*=1.36 × 10^−13^, *BCAS3*), 9p24.2 (rs12236871, *P*=1.48 × 10^−10^, *RFX3*) and 11p15.5 (rs179785, *P*=1.28 × 10^−8^, *KCNQ1*), which contain inflammatory candidate genes. Our results suggest that these loci are most likely related to the progression from hyperuricemia to inflammatory gout, which will provide new insights into the pathogenesis of gout arthritis.

## Abstract (web summary)

Raised serum urate levels are a risk factor for gout, a common form of inflammatory arthritis. Here Li *et al.* conduct a multistage genome-wide association study in a Han Chinese population and identify three novel loci likely associated with the progression from hyperuricemia to gout.

## Graphical abstract

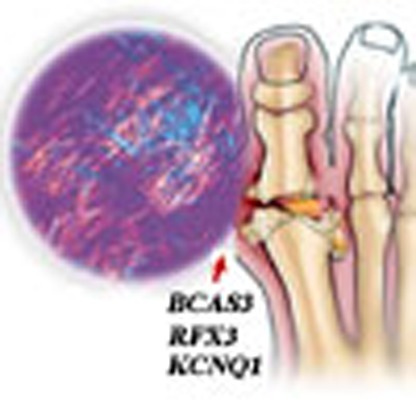

Gout, which is one of the most common types of inflammatory arthritis in men and affects 1–2% of adults in developed countries, results from the deposition of monosodium urate (MSU) crystals in and around the joints^1^,^2^,^3^. Elevated serum urate levels are a key risk factor for gout onset^4^,^5^. However, only ∼10% of people with hyperuricemia develop clinical gout, suggesting that hyperuricemia alone is not sufficient for the development of gout arthritis^6^. Previous genome-wide association studies (GWASs) have identified dozens of loci associated with elevated serum urate levels^7^,^8^,^9^,^10^, whereas little is known about the genetic etiology of the inflammatory response to the MSU crystals^6^. Large, well-defined cohorts of gout and hyperuricemia without gout are required for a GWAS to properly identify genetic loci that control the progression from hyperuricemia to inflammatory gout.

To extend the knowledge of the genetic basis of gout, we conducted GWAS and replication studies in the Han Chinese population using 4,275 clinically ascertained male gout patients and 6,272 healthy male controls in addition to 215 female cases and 541 healthy female controls. Furthermore, 1,644 long-term hyperuricemia patients who had never developed gout were recruited and used to examine whether the newly identified genetic loci are associated with elevated serum urate levels or only with inflammatory gout (Supplementary Fig. 1). We identified three new susceptibility loci that are significantly associated with gout arthritis at 17q23.2 (rs11653176, *P*=1.36 × 10^−13^), 9p24.2 (rs12236871, *P*=1.48 × 10^−10^) and 11p15.5 (rs179785, *P*=1.28 × 10^−8^).

## Results

### Association analyses

During the discovery phase (GWAS stage), we genotyped 1,398 male cases and 1,962 male controls (Supplementary Table 1) using the Affymetrix Axiom Genome-Wide CHB Array. After the quality control (QC), a total of 603,505 single-nucleotide polymorphisms (SNPs) in 1,255 cases and 1,848 controls were retained for further analysis (see Methods). A principal component analysis (PCA)-based analysis was performed to correct for any potential population stratification (Supplementary Fig. 2 and Supplementary Fig. 3, see Methods). Association analyses are performed with logistic regression. The quantile–quantile (Q–Q) and Manhattan plots are shown in Supplementary Fig. 4 and Supplementary Fig. 5a. As expected, the SNP rs2231142 (*P*=4.66 × 10^−10^, Supplementary Table 2), which was previously reported in several GWASs of serum urate levels, hyperuricemia, and gout^7^,^8^,^9^,^10^ (Supplementary Data 1), showed genome-wide significance (*P*<5 × 10^−8^). Because we intended to search for new risk loci, each SNP with a *P*≤5 × 10^−5^ in the discovery stage and with adjacent genes that were not previously reported was selected as a candidate for the follow-up phase I (REP1 stage) study.

In the REP1 stage, among the 79 SNPs with a *P*≤5 × 10^−5^ in the discovery phase, 12 SNPs mapping to the known gout loci (*SLC2A9* and *ABCG2*, Supplementary Data 1) were excluded. With the exception of *BCAS3*, though it (top SNP rs2079742) had already been reported to be associated with serum urate concentrations by Kottgen *et al.*, they didn't provide solid evidence for the association between *BCAS3* and gout (*P*=0.35)^9^. Thus we included the *BCAS3* region as novel loci for gout here. To be noted, our top SNPs (rs9905274 and rs11653176) are in linkage disequilibrium (LD) with rs2079742 (r^2^=0.63 and 0.47, respectively). In each locus, we also filtered for SNPs in tight LD (r^2^>0.5) and retained at least one SNP. Finally, 59 SNPs (three pairs with r^2^>0.5) were selected during the REP1 stage (Supplementary Data 2). We genotyped these SNPs in a cohort of 814 cases and 1,414 controls (Supplementary Table 1, REP1 stage) and found that 5 SNPs showed nominal significance (*P*<0.05) with a direction consistent with the discovery phase. An additional 8 SNPs also had odds ratios (OR) consistent with the direction of the discovery phase with marginal significance in this stage. We chose these 13 SNPs (Supplementary Data 3 and Supplementary Data 4) for the next stage.

**Figure 1:**
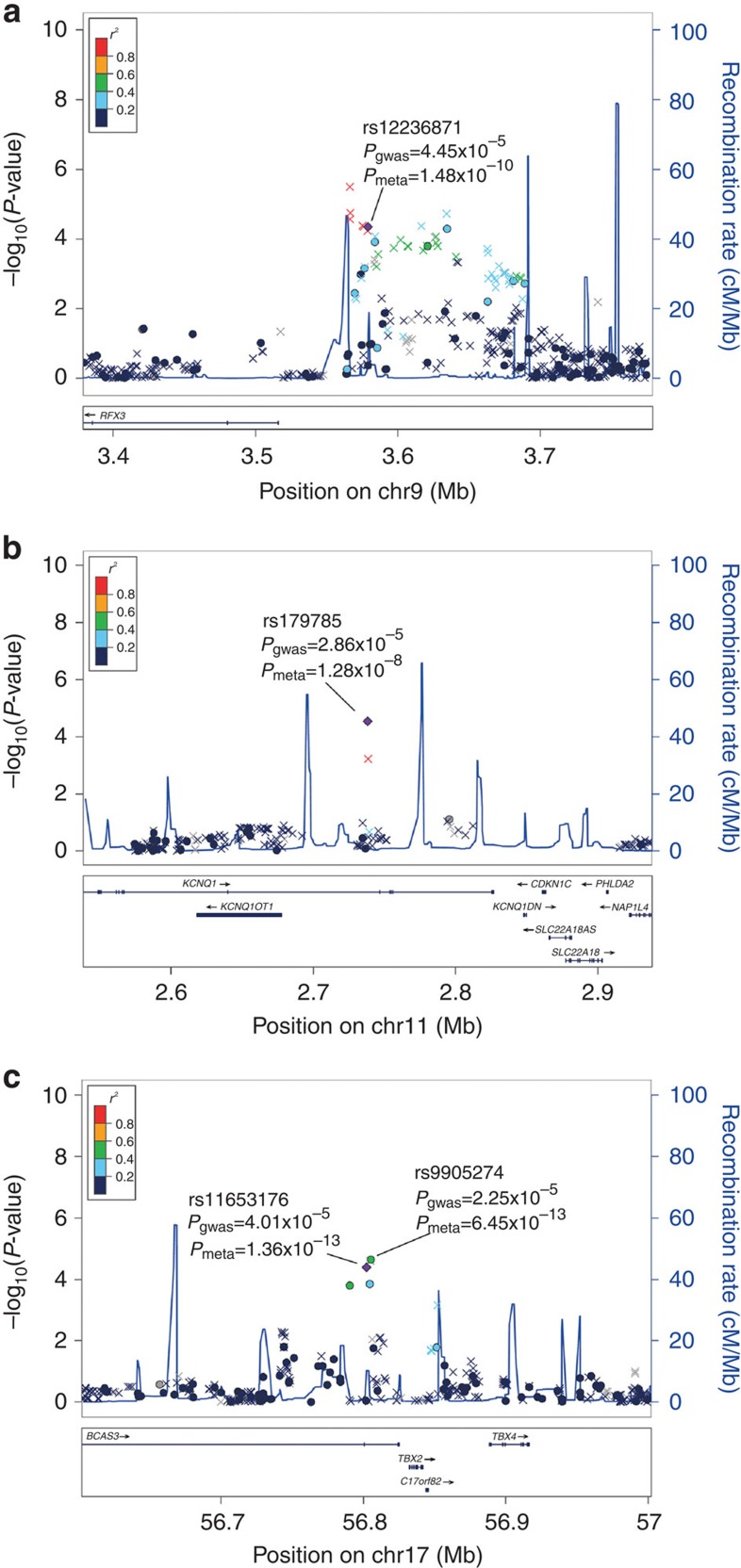
Regional plots of the three loci associated with gout arthritis. (**a**) 9p24.2, (**b**) 11p15.5 and (**c**) 17q23.2. The −log_10_ *P* values are shown for the SNPs within the 200-kb region on either side of the marker SNPs. The index SNP is shown in purple, and the *r*^2^ values of the other SNPs are indicated by colour. The *r*^2^ values are established based on the 1,000 Genome CHB and JPT data (June 2010). The *P*_gwas_ was obtained from the GWAS stage (analysed with logistic regression) and is shown for the genotyped (circle) and imputed (cross) SNPs. The *P*_meta_ was obtained from a meta-analysis combining all of the data sets, including the discovery and replication stages. The genes within the relevant regions are annotated and indicated with arrows.

In the follow-up phase II study (REP2 stage), we genotyped the 13 SNPs in samples recruited from Shandong (882 cases and 1,895 controls, Supplementary Table 1). Four of the SNPs showed nominal significance in this stage, with associated *P* values of <5 × 10^−7^ in the meta-analysis of the GWAS, REP1 and REP2 data (Supplementary Table 3). Then, the 4 SNPs were genotyped in additional independent samples (REP3 stage) from a Northern China data set (996 cases and 786 controls) and a Sichuan data set (328 cases and 329 controls) (Supplementary Table 1). All four SNPs showed a *P*<0.05 in the REP3 stage (Supplementary Table 4). Using a meta-analysis to combine all of the data sets, including the discovery and replication stages, four SNPs, rs11653176 (OR=0.79, *P*=1.36 × 10^−13^, *P*_het_=0.423, I^2^=0%) and rs9905274 (OR=0.79, *P*=6.45 × 10^−13^, *P*_het_=0.260, I^2^=24%, highly linked with rs11653176, *D*′=0.87 and *r*^2^=0.74) at 17q23.2, rs12236871 at 9p24.2 (OR=0.81, *P*=1.48 × 10^−10^, *P*_het_=0.238, *I*^2^=28%) and rs179785 at 11p15.5 (OR=0.82, *P*=1.28 × 10^−8^, *P*_het_=0.123, *I*^2^=45%), reached genome-wide significance (Table 1 and Fig. 1).

### The genome-wide significant SNPs and kidney function

Both the 17q23.2 and 11p15.5 loci have been reported to be associated with measures of renal function and kidney disease in previous GWASs^11^,^12^. As impaired kidney function is one of the main risk factors for gout, we tried to compare the effect estimates for the analyses with or without eGFR (estimated glomerular filtration rate, a measure of kidney function) as a covariate. As we did not collect the serum creatinine data for all individuals throughout the study, we used a subset of sample for whom data on serum creatinine were available for the analysis. In an analysis of 2,473 gout cases and 2,448 controls, we did not see a substantial difference in the effect estimates between with and without adjustment for eGFR (Supplementary Table 5). It suggests the identified genome-wide significant SNPs for gout are more likely to be independent of impaired kidney function.

### The genome-wide significant SNPs and hyperuricemia

To further investigate whether the four genome-wide significant SNPs were either associated with or independent of hyperuricemia, we additionally analysed a cohort with 1,644 long-term hyperuricemia patients (Supplementary Table 1) whose serum urate levels were higher than 420 μmol l^−1^ and who had experienced hyperuricemia for >10 years without any treatment to reduce urate but had not developed gout when the samples were collected. The analysis of the 1,644 special hyperuricemia patients and healthy controls in this study showed that the four SNPs located in the three loci are not associated with hyperuricemia (*P*>0.05, Table 2), but their allele frequencies were significantly different between the gout and the special hyperuricemia groups (*P*<0.05, Table 2). Therefore, our results suggest that these genetic associations should be linked to mechanisms other than hyperuricemia in gout.

### The genome-wide significant SNPs and female gout

Because the molecular mechanism associated with female gout is considered to be different from that of male gout, we also analysed the four SNPs in a small female cohort of 215 cases and 541 controls (Supplementary Table 6), and one SNP (rs12236871, *P*=0.005) showed nominal significance (*P*<0.05).

### Assessment of the regulatory potential for the novel loci

To explore the potential implications and epigenetic profile of the association signals, we queried the index SNPs and their proxies (*r*^2^>0.8) based on LD in the 1,000 Genomes Project ASI data set using HaploReg v2^13^. Some signs of regulatory activity (promoter, enhancer and DNase hypersensitivity sites, binding proteins and motifs changed) were observed for the associated SNPs and their surrogates, indicating a possible effect on transcription for these loci (Supplementary Table 7).

### Previously reported risk loci for hyperuricemia or gout

We also verified the previously identified loci in hyperuricemia or gout (Supplementary Data 1,64 SNPs at 35 loci with a *P*<5 × 10^−8^) in the NHGRI GWAS catalog (as of 03/25/14). Ten of the 64 SNPs are with low frequency (<1%) in our samples, 54 SNPs at 28 loci were kept for the association analyses of gout and serum urate levels (the gout samples) in our discovery data set (Supplementary Data 5). For gout, 18 SNPs at 8 loci showed significance (*P*<0.05), and all are with consistent directions of the previous reports, except for rs4698014 that is uncertain due to the OR was not given in the previous report. For serum urate levels, six SNPs at four loci showed significance, and all (except for rs729761) are with consistent directions of the previous reports.

## Discussion

In this multistage GWAS of gout arthritis, we identified four SNPs that were significantly associated (*P*<5 × 10^−8^) with gout risk in Chinese population. The top two signal SNPs (rs11653176 and rs9905274) are localized in the intron of *BCAS3* (breast carcinoma amplified sequence 3). *BCAS3* is an estrogen-induced transcriptional co-activator that is overexpressed in breast cancer^14^,^15^, and it is associated with tumour grade and proliferation^16^. In a previous GWAS of serum urate concentrations in >140,000 individuals of European ancestry, *BCAS3* (rs2079742) was discovered and gained further support from data of non-European ancestry populations, but not to be significant (Indian,*n*=8,340, African-American,*n*=5,820 and Japanese, *n*=15,286)^9^. In our data, the effect size of rs2079742 on serum urate was comparable to previous estimates from Asian populations, but due to the small sample size, we did not observe significant association. And the SNPs rs11653176 and rs9905274 did not show a significant association with hyperuricemia, and with serum urate levels in the combined hyperuricemia and controls, either. The fact that many of the gout patients were treated may also influence the results for urate levels association analyses. Another nearby gene, *TBX2*, which is located ∼7 kb downstream of *BCAS3*, could be a candidate in this region as well. TBX2 has been reported to modulate the expression of IFN-gamma^17^. A previous study indicated that the MSU crystals alone did not induce NO production in murine macrophages, while a synergistic effect on the level of iNOS expression and NO generation was observed in cells exposed to MSU crystals in combination with IFN-γ^18^. Therefore, it is possible that TBX may be involved in gout development by regulating IFN-γ. Thus, both *BCAS3* and *TBX2* are the most likely genes involved in the association observed at this locus.

The third identified SNP, rs12236871, mapped to 53-kb upstream of *RFX3* (regulatory factor 3). This association signal was also observed in the female cohort. RFX3 is a transcription factor involved in the control of ciliogenesis. It is expressed in the ciliated ependymal cells of the subcommissural organ, choroid plexuses and ventricular walls. RFX3 has also been found to be necessary for the differentiation and function of mature beta-cells and regulates GCK expression and mature beta-cell function by binding to its promoter^19^. Interestingly, GCKR, another regulatory protein of GCK, was reported to be associated with serum uric acid levels in individuals of European descent^8^ and with gout in the Han Chinese^20^.

The fourth SNP, rs179785, resides within the intron region of *KCNQ1*, the potassium voltage-gated channel (KQT-like subfamily) member 1 gene. *KCNQ1* is expressed in the mid- to late-proximal tubule of the kidney and along the entire gastrointestinal tract. A recent study indicated that *KCNQ1* is involved in mouse and human gastrointestinal cancer development, and the loss of *KCNQ1* in mice leads to alterations in the genes involved in innate immune responses^21^.

Notably, *KCNQ1* has shown a strong association with type 2 diabetes (T2D) in several GWASs^22^,^23^,^24^. Several studies found that common variants of *KCNQ1* may also confer susceptibility to diabetic nephropathy, especially in East Asian populations^25^,^26^,^27^. To avoid the influence of T2D, in the follow-up stages of our study, all of the cases and controls were filtered for diabetes. Besides, the SNP (rs179785) is in low/moderate linkage disequilibrium with the reported T2D associated SNPs in the 1,000 Genome Asian samples (*r*^2^=0.001 to 0.044 and D'=0.038 to 0.421, Supplementary Table 8). Therefore, the association with gout identified here should be independent of T2D.

In this study, we recruited 4,275 male gout patients and 215 female cases, but this sample size was still limited, especially for the separate analysis for each stage. Not more than 1,500 cases were included for each stage. The discovery sample sizes used in this study can detect the effect sizes of median risks (ORs range from 1.35 to 1.50) for common alleles (frequencies range from 0.10 to 0.75) at the significance threshold used for follow-up (*P*<5 × 10^−5^) with the power greater than 80% (Supplementary Table 9). However, our sample size for each stage may still be underpowered for detecting small risks.

In conclusion, the combined multiple-stage analysis identified three new loci located in 17q23.2, 9p24.2 and 11p15.5 that are associated with gout but not with hyperuricemia without gout in the Han Chinese population. The candidate genes located in these regions likely play important roles in the etiology of gout arthritis but not hyperuricemia. However, further validations, especially functional experiments, are suggested. Our identification of these new common genetic risk variants of gout arthritis provides new insights into the pathogenesis of this disease.

## Methods

### Ethics

The sample collection and the clinical information regarding the subjects were undertaken following informed consent and approval by the relevant ethics review board at the Affiliated Hospital of Qingdao University, in accordance with the tenets of the Declaration of Helsinki.

### Subjects

All of the gout patients analysed in the study were interviewed by endocrinologists and diagnosed according to the American College of Rheumatology criteria for gout^28^. All of the cases in the discovery and validation stages were recruited using the same diagnostic criteria. The healthy controls were attained via site survey. Practice lists of healthy controls were screened for potentially suitable subjects by excluding those with hyperuricemia, diabetes, cancer and other arthritis-related illnesses.

The discovery phase included 1,255 male cases recruited from Shandong Province and 1,848 normal male controls recruited from the northern China area, including Shandong, Shanxi, Hebei, and Beijing.

Follow-up stages I and II (REP1 and REP2) included 814 male cases and 1,414 healthy male controls and 882 male cases and 1,895 healthy male controls, respectively. All of the samples were recruited from Shandong Province. Follow-up stage III (REP3) included two data sets: the Northern China sample set, which consisted of 996 male cases and 786 healthy male controls, and the Sichuan Province sample set, which consisted of 328 male cases and 329 healthy male controls.

The female cohort was comprised of 215 cases and 541 healthy controls. We recruited 1,644 hyperuricemia patients who had experienced hyperuricemia for more than 10 years without urate-lowering therapy but who had never developed gout. The serum urate level of the males and postmenopausal females was greater than 420 μmol l^−1^, while it was greater than 360 μmol l^−1^ in the premenopausal females.

The sample descriptions can be found in Supplementary Table 1. All of the subjects in the replication stage were unrelated.

### Quality control (QC) of the GWAS data set

A total of 3,360 arrays were used in the GWAS, including 25 arrays for the designed duplication of the randomly selected samples. A dish QC (DQC) value greater than 0.82 was set as the primary quality-control step. A total of 94 samples were excluded from further data analyses due to DQC failure. A total of 6 samples were excluded because the self-reported genders did not match the genotyped genders. The members with a lower experimental quality in the duplicate pair, which were genotyped as an internal control for the experimental QC, were excluded from the analysis (*n*=25). The genotype data were generated using Axiom Genotyping Algorithm v1 (Axiom GT1). For the sample filtering, arrays with generated genotypes for <95% of the loci were excluded (*n*=40). The heterozygosity rates were calculated and deviations of more than 6 s.d. from the mean were excluded (no samples were excluded). PLINK's identity by descent analysis was used to detect the hidden relatedness. When pairs of individuals had a PI_HAT>0.25, the member of the pair with the lower call rate was excluded from the analysis (*n*=92); 1,255 cases and 1,848 controls were retained for further analyses. For the SNP filtering (after sample filtering), SNPs with call rates <95% in the samples were removed (*n*=36,349). SNPs with a minor allele frequency<3% (*n*=12,267) or SNPs that deviated significantly (*P*≤1 × 10^−5^) from Hardy–Weinberg equilibrium in the controls (*n*=15,707) were also excluded. A total of 603,697 SNPs passed the quality criteria and were used in the subsequent analyses.

### Population stratification analysis

The population stratification was assessed using a PCA-based method implemented in the software package EIGENSTRAT^29^. First, we performed a PCA of a combination of our samples and 270 reference HapMap samples to evaluate the population structure of the samples. The first two eigenvectors are plotted in Supplementary Fig. 2. We performed a second PCA for the discovery set for the population stratification correction (Supplementary Fig. 3). A total of 20 principal components were generated for the correction.

### SNP genotyping in the replication phases

The genotyping for replication I was performed using the iPLEX platform (Sequenom, San Diego, CA), and replications II and III were performed using the ligation detection reaction method^30^,^31^, with technical support from the Shanghai Biowing Applied Biotechnology Company.

### Statistical analysis

For gout, logistic regression was used to test the association of a single SNP using PLINK (http://pngu.mgh.harvard.edu/~purcell/plink/)^32^, and the 20 principal components were used as covariates in the association analysis to correct for the population stratification. After adjustment, little stratification was observed (*λ*=1.058, *λ*_1,000_=1.039, standardized to a sample size of 1,000). A fixed-effects model with inverse variance weighting was used in the meta-analysis. Heterogeneity across the data sets was evaluated using Cochran's Q test, and the *I*^2^ index was used to quantify the degree of heterogeneity. For serum urate levels, the phenotypes were normalized to a standard normal distribution for further association analysis, the association analyses were conducted using SNPTEST^33^. A Manhattan plot of the −log_10_ (*P* values) was generated using Haploview^34^. Ungenotyped SNPs of the autosomes were imputed in the GWAS discovery samples using SHAPEIT 2.0 (http://www.shapeit.fr/)^35^ (phasing step), IMPUTE2 (, http://mathgen.stats.ox.ac.uk/impute/impute_v2.html)^36^ (imputation step) and the haplotype information from the 1,000 Genomes Project (Phase I integrated variant set across all 1,092 individuals, v2, March 2012; http://www.1000genomes.org/; Supplementary Fig. 5b). The online tool HaploReg (http://hapmap.ncbi.nlm.nih.gov/) was used to explore chromatin states, conservation and regulatory motif alterations of the associated loci^13^. The input for HaploReg consisted of the six index SNPs, and the *r^2^* threshold was set at 0.8 (based on the 1,000G Phase 1 ASI population for the LD calculation). Regional plots were generated using the online tool LocusZoom 1.2 37 (http://csg.sph.umich.edu/locuszoom/). Power analysis was conducted using the genetic power calculator at risk allele frequency ranges from 0.05 to 0.85 and OR ranges from 1.10 to 1.50 38.

## Additional information

**How to cite this article:** Li, C. *et al.* Genome-wide association analysis identifies three new risk loci for gout arthritis in Han Chinese. *Nat. Commun.* 6:7041 doi: 10.1038/ncomms8041 (2015).

## Supplementary Material

Supplementary Information Supplementary Figures 1-5, Supplementary Tables 1-9

Supplementary Data 1 The loci identified in the previous GWASs for serum uric acid levels, urate levels and gout.

Supplementary Data 2 Results of the discovery phase (GWAS) for the 59 replication SNPs.

Supplementary Data 3 Results of the follow-up phase I (REP 1) for the 59 replication SNPs.

Supplementary Data 4 Results of the GWAS-REP1 meta-analysis for the 59 replication SNPs.

Supplementary Data 5 Results for the association analyses of gout and serum urate levels (the gout samples) in our stage 1 dataset for the previously reported SNPs.

## Figures and Tables

**Table 1:** Results for the four genome-wide significant SNPs.

**Chromosome**	**SNP**	**Position**	**A1/Freq.**	**GWAS (1,255 cases versus 1,848 controls)**		**REP1 (814 cases versus 1,414 controls)**		**REP2 (882 cases versus 1,895 controls)**		**REP3 (1,324 cases versus 1,115 controls)**		**Meta-analysis of GWAS and REPs**			
				P*	OR*	P*	OR*	P*	OR*	P*	OR*	***P***†	**OR**†	**Q**	**I**
9	rs12236871	3579117	G/0.411	4.45 × 10^−5^	0.67	8.08 × 10^−3^	0.84	6.47 × 10^−4^	0.82	1.46 × 10^−3^	0.83	1.48 × 10^−10^	0.81	0.238	28
11	rs179785	2738095	G/0.511	2.86 × 10^−5^	0.66	3.95 × 10^−4^	0.79	3.27 × 10^−2^	0.88	2.79 × 10^−2^	0.87	1.28 × 10^−8^	0.82	0.123	45
17	rs11653176	56802151	T/0.472	4.01 × 10^−5^	0.68	2.97 × 10^−4^	0.80	1.04 × 10^−5^	0.77	2.38 × 10^−3^	0.84	1.36 × 10^−13^	0.79	0.423	0
17	rs9905274	56805223	T/0.474	2.25 × 10^−5^	0.66	1.81 × 10^−2^	0.86	2.56 × 10^−5^	0.78	7.38 × 10^−5^	0.79	6.45 × 10^−13^	0.79	0.260	24

GWAS, genome-wide association studies; SNP, single-nucleotide polymorphism; OR, odds ratio;

Shown are the discovery (GWAS), replications (REP1, REP2 and REP3) and meta-analysis results for gout cases versus controls. Position, based on hg18; A1, minor allele for the whole sample; The minor allele was the coded allele; Freq., the frequency of A1 in the controls; OR, odds ratio; *P*, *P*-value; Q, *P*-value for Cochrane's Q statistic; *I*^2^, *I*^2^ heterogeneity index (0–100).

^*^The OR and *P* values are based on the logistic regression.

^†^The OR and *P* values are based on the meta-analysis under fixed-effects model.

**Table 2:** Comparisons of the four genome-wide significant SNPs in the HU versus control group and gout versus HU group.

**Chromosome**	**SNP**	**Position**	**A1**	**Freq. in the gout group**	**Freq. in the HU group**	**Freq. in the control group**	**HU versus controls**		**Gout versus HU**	
							***P***	**OR**	***P***	**OR**
9	rs12236871	357,9117	G	0.367	0.398	0.411	0.250	0.95	4.39E-03	0.88
11	rs179785	273,8095	G	0.454	0.527	0.526	0.933	1.00	1.34E-10	0.75
17	rs11653176	56,802,151	T	0.411	0.461	0.469	0.456	0.97	4.95E-06	0.81
17	rs9905274	56,805,223	T	0.427	0.458	0.472	0.209	0.95	4.97E-03	0.88

HU, hyperuricemia; SNP, single-nucleotide polymorphisms; OR, odds ratio;

Position, based on hg18; A1, minor allele for the whole sample; OR, odds ratio; *P*, *P*-value. The gout group includes 2,951 gout cases from GWAS, REP1 and REP2 stages, the HU (hyperuricemia) group includes 1,644 hyperuricemia cases, and the control group 3,309 controls from REP1 and REP2 stages. The OR and *P* values are based on the logistic regression.
