# Supplementary material for: Genome-wide association analysis identifies three new risk loci for gout arthritis in Han Chinese
Source: Nat Commun. 2015 May 13;6:7041. doi: 10.1038/ncomms8041 (PMC4479022; doi:10.1038/ncomms8041)
Supplement: Supplementary Information — Supplementary Figures 1-5, Supplementary Tables 1-9 [file ncomms8041-s1.pdf]

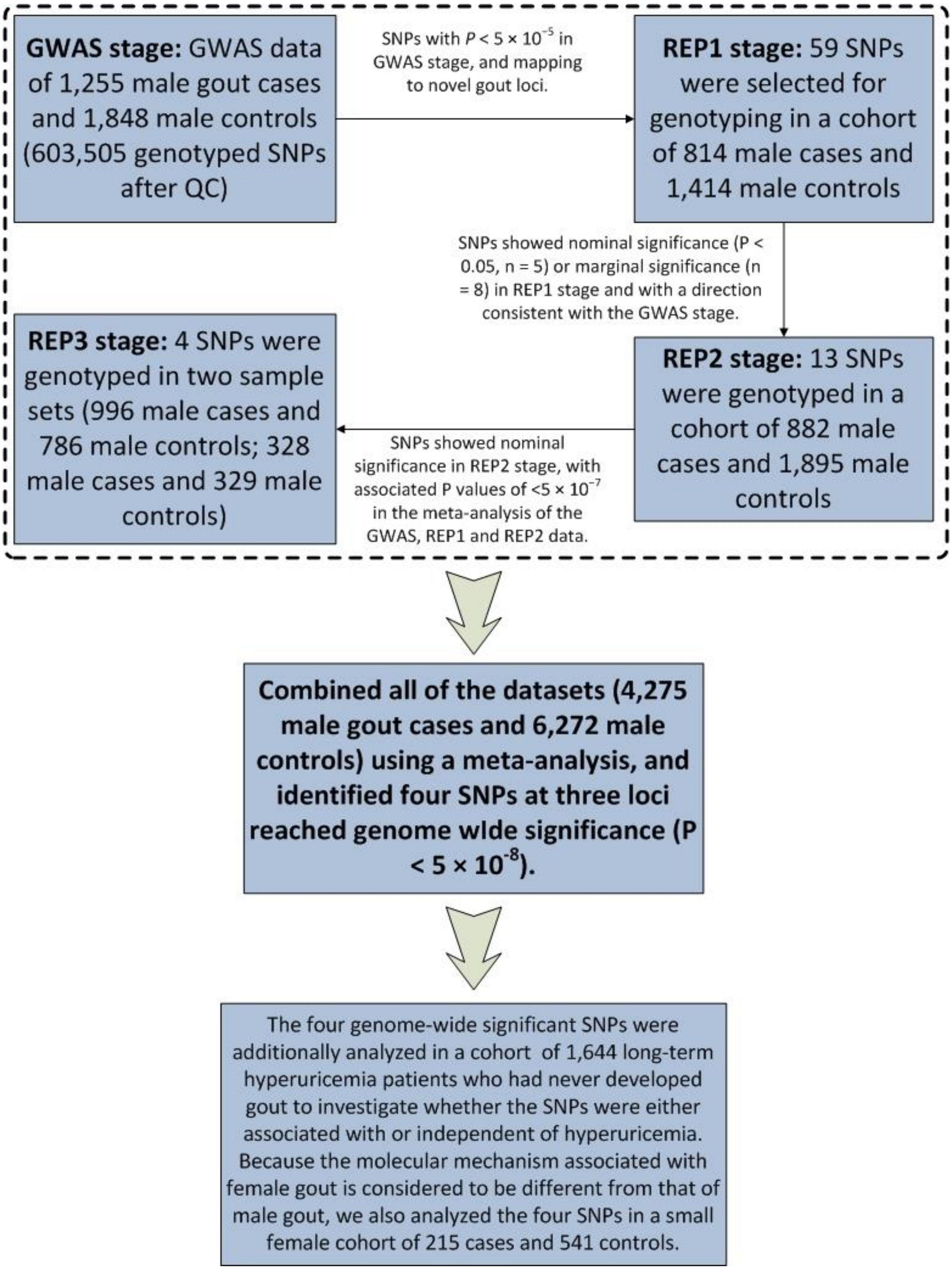

Supplementary Figure 1. Study design of a multi-stage GWAS of gout.

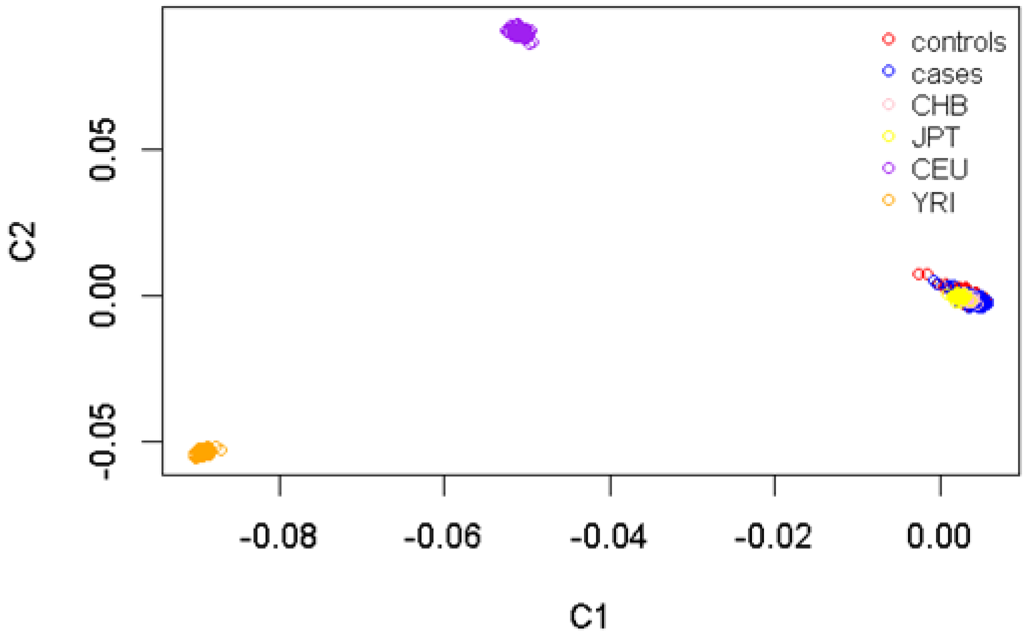

Supplementary Figure 2. Plot of the first two principal components from the analysis of the genome-wide study (after QC) combined with HapMap data. The samples' disease status and the ethnicity of the HapMap samples are indicated by color. Briefly, Controls are red circles (n = 1,848); Cases are blue circles (n = 1,255); HapMap Chinese (CHB) samples are pink circles (n = 45), Japanese (JPT) samples are yellow circles (n = 45), European (CEU) samples are purple circles (n = 90) and African (YRI) samples are orange circles (n = 90).

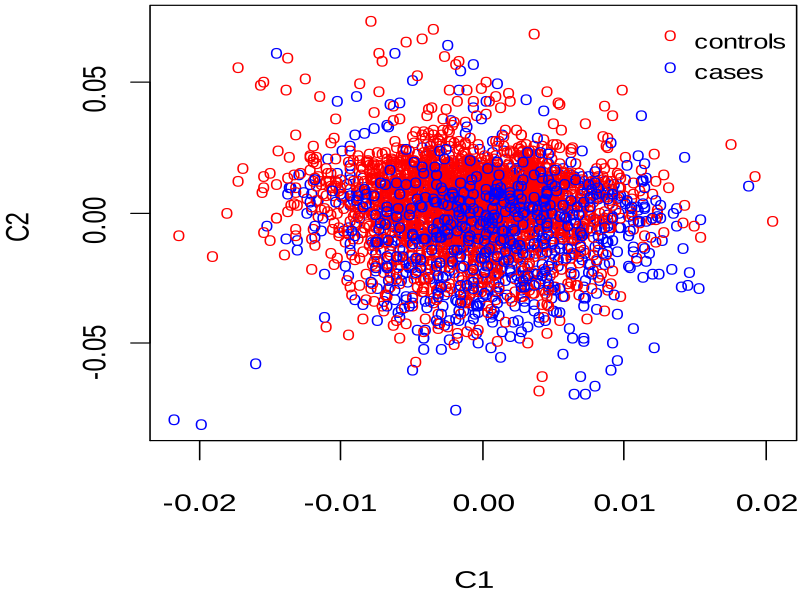

Supplementary Figure 3. Plot of the first two principal components from the analysis of the genome-wide study (after QC). Controls are red circles (n = 1,848) and Cases are blue circles (n = 1,255).

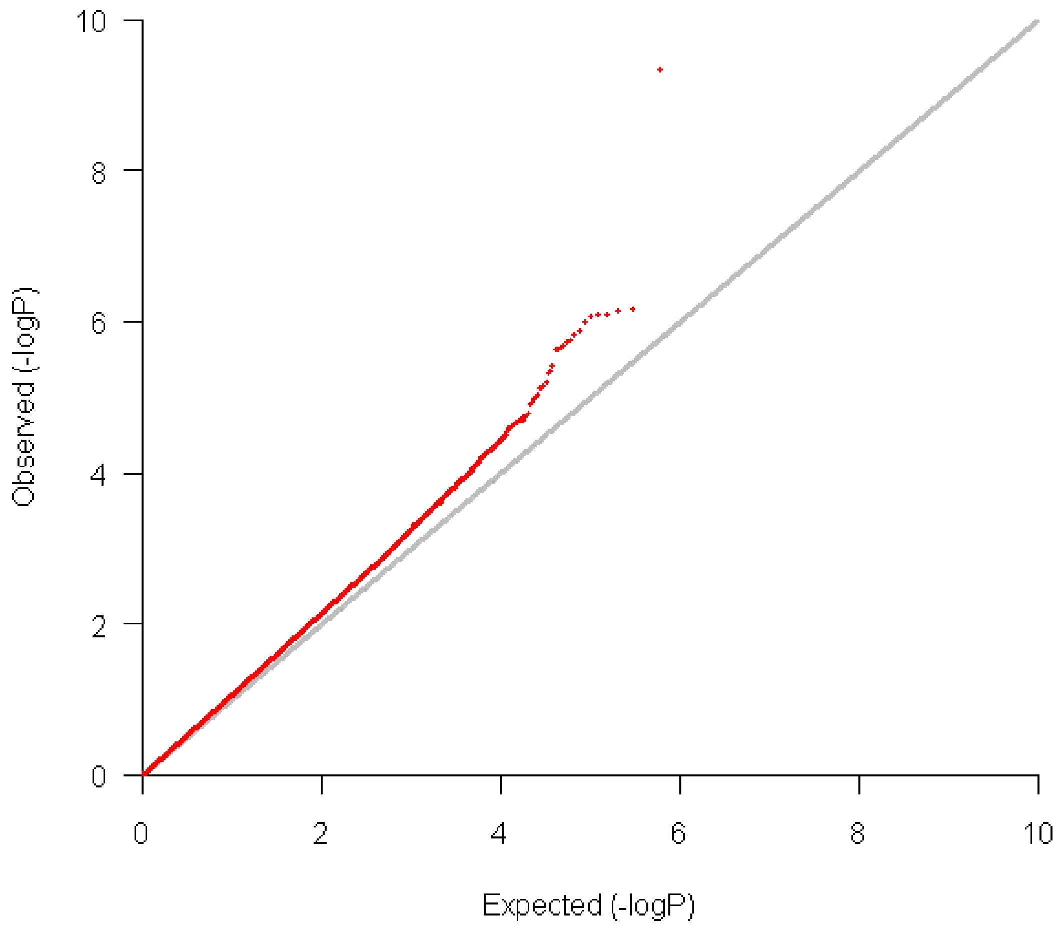

Supplementary Figure 4. Quantile-Quantile (Q-Q) plot of the discovery data.

a) Genotyped

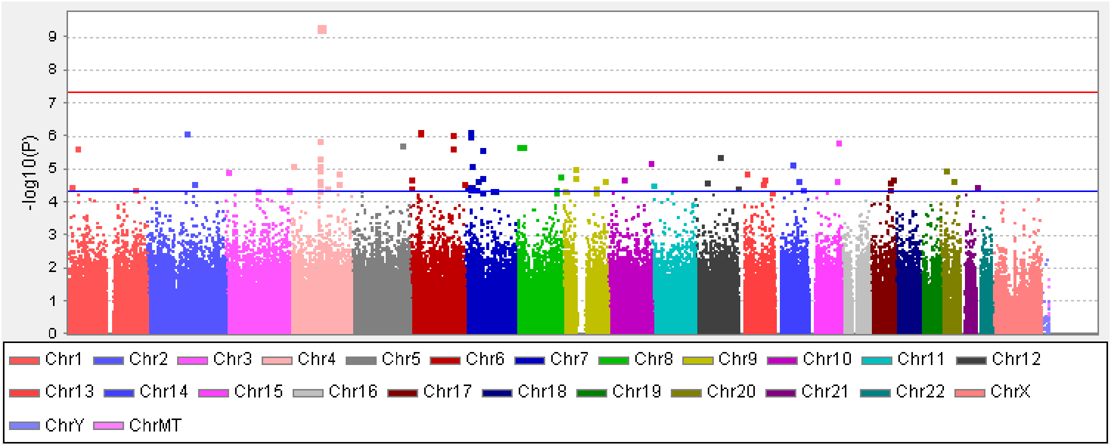

b) Genotyped and Imputed (autosomes)

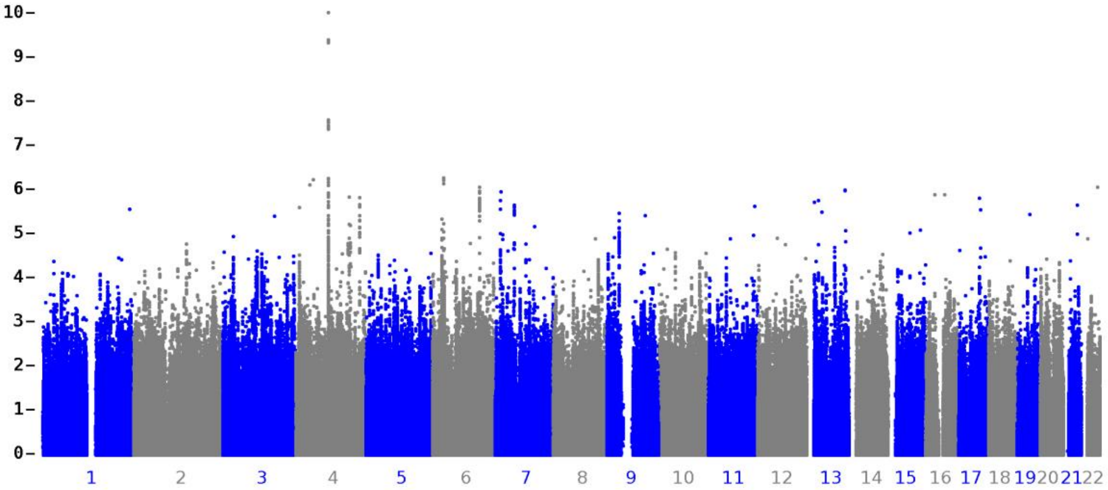

Supplementary Figure 5. Plot of genome-wide association result for the discovery data. Chromosomes are delineated by alternating colors, as labeled on the x-axis. The y-axis shows the –log10 P-values (using logistic regression).

Supplementary Table 1. Description of the discovery and replication phases samples.

	N	Location	%Female	Age (years±s.d.)	Uric Acid (umol l^{-1}±s.d.)
**Discovery Phase**					
Cases	1255	Shandong Province	0	52.9±13.0	458.7±107.6
Controls	1848	Northern China[a]	0	53.9±7.92	-
**Replication Phase 1**					
Cases	814	Shandong Province	0	49.4±12.8	468.8±110.2
Controls	1414	Shandong Province	0	53.4±15.3	290.6±48.5
**Replication Phase 2**					
Cases	882	Shandong Province	0	50.6±13.7	463.7±110.6
Controls	1895	Shandong Province	0	49.7±13.1	294.1±46.6
**Replication Phase 3**					
Cases	996	Northern China[a]	0	51.5±13.8	458.3±126.8
Controls	786	Northern China[a]	0	47.9±14.2	290.1±50.9
Cases	328	Sichuan Province	0	52.4±14.2	470.6±135.4
Controls	329	Sichuan Province	0	47.2±10.6	301.9±41.6
**Female cohort**					

	N	Location	%Female	Age (years±s.d.)	Uric Acid (umol l^{-1}±s.d.)
Cases	215	Shandong Province (Mainly)	100	62.5±11.2	407.9±120.2
Controls	541	Shandong Province (Mainly)	100	64.1±11.0	235.4±39.3
**Hyperuricemia cohort**					
	1644	Shandong Province (Mainly)	11.5	50.7±15.5	474.9±56.2

[a]Northern China: Shandong, Heilongjiang, Shanxi, Hebei, and Beijing.

Supplementary Table 2. Results for the SNPs shown P<5e-5 in the discovery phase (GWAS) within the loci identified in the previous GWASs.

Chr.	SNP	Position	A1	Freq.	OR[a]	P[a]
4	rs11722228	9524839	T	0.274	1.53	7.63E-06
4	rs2728131	89143368	T	0.244	1.56	2.42E-05
4	rs2725234	89151296	T	0.242	1.59	1.00E-05
4	rs2725211	89189399	T	0.240	1.56	2.11E-05
4	rs34455506	89243244	A	0.218	0.57	2.73E-05
4	rs2725263	89245452	A	0.447	0.64	2.41E-05
4	rs34472643	89248890	A	0.234	0.56	4.48E-06
4	rs12505410	89249865	G	0.302	0.57	1.36E-06
4	rs2231146	89258524	C	0.191	0.54	7.49E-06
4	rs2231142	89271347	T	0.309	1.82	4.66E-10
4	chr4_89280826	89280826	G	0.170	0.51	4.71E-06
4	rs2725252	89280934	A	0.396	0.67	4.54E-05
17	rs11653176	56802151	T	0.475	0.68	4.01E-05
17	rs9905274	56805223	T	0.479	0.67	2.25E-05

Position, based on hg18; A1, minor allele; Freq., frequency of A1 in controls; OR, odds ratio; The minor allele was the coded allele; P, P value.

[a]The OR and P values are PCA-adjusted (using logistic regression).

Supplementary Table 3. Results of the follow-up phase II (REP 2) and GWAS-REP1-REP2 meta-analysis for the 13 replication SNPs.

Chr.	SNP	Position	A1	A2	REP2 (882 cases and 1895 controls)				GWAS-REP1-REP2-META			
					F_A	F_U	P^a	OR^a	P^b	OR^b	Q	I
1	rs116862989	206420078	G	T	0.188	0.182	6.06E-01	1.04	2.53E-03	1.17	0.006	80.38
3	rs76159432	3947376	C	T	0.068	0.075	4.00E-01	0.90	2.45E-02	1.20	0.000	88.29
3	rs76289943	194709817	A	G	0.093	0.094	9.71E-01	1.00	7.94E-03	1.20	0.003	82.52
7	rs4721507	16510836	G	C	0.174	0.174	9.73E-01	1.00	2.72E-03	0.85	0.000	88.55
7	rs62465767	51635485	A	G	0.176	0.177	8.78E-01	0.99	1.33E-02	1.14	0.002	83.54
7	rs11764153	92861504	T	C	0.466	0.469	8.58E-01	0.99	7.34E-03	0.90	0.004	82.25
8	rs6992081	21304636	G	A	0.064	0.051	6.23E-02	1.28	5.57E-05	1.41	0.004	81.61
9	rs12236871	3579117	G	A	0.361	0.409	6.47E-04	0.82	2.39E-08	0.80	0.126	51.67
11	rs179785	2738095	G	A	0.503	0.535	3.27E-02	0.88	8.91E-08	0.80	0.050	66.71
13	rs6562946	75664604	C	T	0.463	0.425	1.31E-02	1.17	1.01E-05	1.19	0.030	71.53
14	rs3784099	67819680	A	G	0.139	0.143	6.92E-01	0.97	7.61E-05	0.80	0.003	82.87
17	rs11653176	56802151	T	C	0.405	0.469	1.04E-05	0.77	6.30E-12	0.76	0.350	4.83
17	rs9905274	56805223	T	C	0.404	0.466	2.56E-05	0.78	1.98E-09	0.79	0.073	61.88

SD, Shan Dong; SC, Si Chuan; Position, based on hg18; A1, minor allele; A2, major allele; F_A, frequency of the minor allele in cases; F_U, frequency of the minor allele in controls; OR, odds ratio; The minor allele was the coded allele; P, P value; Q, p-value for Cochrane's Q statistic; I^2, I^2 heterogeneity index (0-100). [a]The OR and P values are based on the logistic regression. [b]The OR and P values are based on the meta-analysis under fixed-effects model.

Supplementary Table 4. Results of the follow-up phase III (REP 3) for the 4 replication SNPs.

Chr.	SNP	Position	A1	A2	REP3-META				REP3-NC (996 cases and 786 controls)				REP3-SC (328 cases and 329 controls)			
					P[a]	OR[a]	Q	I	F_A	F_U	P[b]	OR[b]	F_A	F_U	P[b]	OR[b]
9	rs12236871	3579117	G	A	1.46E-03	0.83	0.282	13.74	0.386	0.422	0.031	0.86	0.343	0.412	0.010	0.74
11	rs179785	2738095	G	A	2.79E-02	0.87	0.683	0	0.504	0.535	0.111	0.88	0.502	0.546	0.116	0.84
17	rs11653176	56802151	T	C	2.38E-03	0.84	0.786	0	0.441	0.483	0.015	0.85	0.442	0.492	0.067	0.82
17	rs9905274	56805223	T	C	7.38E-05	0.79	0.857	0	0.429	0.489	5.07E-04	0.79	0.437	0.490	0.055	0.81

NC, Northern China; SC, Si Chuan; Position, based on hg18; A1, minor allele; A2, major allele; F_A, frequency of the minor allele in cases; F_U, frequency of the minor allele in controls; OR, odds ratio; The minor allele was the coded allele; P, P value; Q, p-value for Cochrane's Q statistic; I^2, I^2 heterogeneity index (0-100). [a]The OR and P values are based on the meta-analysis under fixed-effects model. [b]The OR and P values are based on the logistic regression.

Supplementary Table 5. Association results without and with adjustment for eGFR in a subset sample for the four genome-wide significant SNPs.

Chr.	SNP	Position	A1	Without adjustment for eGFR		With adjustment for eGFR	
				OR	P	OR	P
9	rs12236871	3579117	G	0.83	1.46E-05	0.83	2.01E-05
11	rs179785	2738095	G	0.77	1.32E-09	0.76	2.57E-09
17	rs11653176	56802151	T	0.79	7.63E-09	0.78	1.69E-08
17	rs9905274	56805223	T	0.82	3.64E-06	0.81	2.01E-06

Position, based on hg18; A1, minor allele; OR, odds ratio; The minor allele was the coded allele; P, P value; eGFR, estimated glomerular filtration rate. The OR and P values are based on the logistic regression.

Supplementary Table 6. Results of the female cohort for the four genome-wide significant SNPs.

Chr.	SNP	Position	A1	F_A	F_U	A2	P	OR
9	rs12236871	3579117	G	0.357	0.436	A	0.005	0.72
11	rs179785	2738095	G	0.497	0.505	A	0.777	0.97
17	rs11653176	56802151	T	0.491	0.492	C	0.975	1.00
17	rs9905274	56805223	T	0.483	0.481	C	0.931	1.01

Position, based on hg18; A1, minor allele; A2, major allele; F_A, frequency of the minor allele in cases; F_U, frequency of the minor allele in controls; OR, odds ratio; The minor allele was the coded allele; P, P value. The OR and P values are based on the logistic regression.

Supplementary Table 7. Summary of genomic annotation by HaploReg v2 for the genome wide significant loci.

CHR	SNP	LD		Promoter	Enhancer	DNAse	Proteins bound	Motifs changed
		r^2	D'					
9	rs2184239	0.85	0.95	CD34.MBP1562	8 cell types	H1-hESC,CD34+_Mobilized,WERI-Rb-1	CTCF	4 altered motifs
9	rs12238832	0.89	0.96	CD34.MBP1562	8 cell types	6 cell types	CTCF	GR
9	rs10972556	0.89	0.96		6 cell types			Egr-1
9	rs10972572	0.96	0.99		CD34.C			7 altered motifs
9	rs10972574	0.96	0.99		CD34.C			AP-1,CEBPB,PEBP
9	rs10972576	0.97	0.99					DMRT2,HDAC2,RXR::LXR
9	rs7040793	0.99	1.00					
**9**	**rs12236871**	**1.00**	**1.00**					**LUN-1,PLZF**
**11**	**rs179785**	**1.00**	**1.00**		**4 cell types**			**10 altered motifs**
11	rs179784	0.96	0.99		5 cell types	iPS		4 altered motifs
**17**	**rs11653176**	**1.00**	**1.00**		**4 cell types**	**HMEC,Caco-2**		**4 altered motifs**
**17**	**rs9905274**	**1.00**	**1.00**					**Egr-1,Mtf1,Pax-4**

CHR, chromosome; the LD information were derived from the 1000 Genomes Project ASI data for the associated SNP (marked in bold) and its surrogates. Promoter, Enhancer, DNAse, Proteins bound and Motifs changed demonstrated evidence of histone modifications, DNase hypersensitivity sites or transcription factor occupancy as shown by the HaploReg v2 analysis.

Supplementary Table 8. LD of the gout associated SNP and the reported T2D associated SNPs in KCNQ1 region.

Gout associated SNP	Urate associated SNP	Distance	r^2	D’
rs179785	rs231362	90048	0.044	0.421
rs179785	rs2237897	77027	0.033	0.189
rs179785	rs163184	65550	0.009	0.103
rs179785	rs163182	62697	0.008	0.106
rs179785	rs2237892	58232	0.005	0.075
rs179785	rs8181588	50022	0.002	0.048
rs179785	rs2237895	75675	0.001	0.038

The r2 and D’ values were estimated from the 1000Genome Asian dataset.

Supplementary Table 9. Power Analyses at P < 5e-5 for the GWAS discovery stage.

**OR**	**0.05**	**0.10**	**0.20**	**0.30**	**0.40**	**0.55**	**0.75**	**0.85**
**1.10**	0%	0%	1%	1%	1%	1%	1%	0%
**1.20**	1%	3%	11%	18%	22%	20%	8%	3%
**1.25**	2%	9%	28%	41%	46%	41%	18%	6%
**1.30**	4%	19%	50%	66%	70%	64%	32%	11%
**1.35**	9%	34%	**72%**	**84%**	**87%**	**81%**	47%	19%
**1.40**	16%	52%	**87%**	**94%**	**95%**	**92%**	61%	27%
**1.50**	38%	**82%**	**98%**	**100%**	**100%**	**99%**	**82%**	45%

Power figures at representative and relevant ORs (ORs of 1.10 to 1.50) and allele frequencies (0.05 to 0.85) are displayed for the Stage 1 GWAS (discovery) analysis. The powers over 80% were indicated in bold.
